# The trade-off between design fixation and quality: Physical objects or multiperspective pictures?

**DOI:** 10.1371/journal.pone.0254933

**Published:** 2021-07-21

**Authors:** Danni Shen, Xuelin Yao, Defu Bao

**Affiliations:** Art and Design Institute, Zhejiang Sci-Tech University, Hangzhou, Zhejiang, China; Iowa State University, UNITED STATES

## Abstract

Physical objects and their pictures are two main kinds of design stimuli of creative activity, which can improve design quality but may induce design fixation. Previous studies are focused on the case where participants face a single picture, and their design stimulus may be incomplete as compared with the participants facing objects. To fully explore the influence of physical and pictorial examples on design novices, we investigated design fixation and design quality when they were provided with multiperspective pictures having information remarkably similar to physical objects. Specifically, two novice groups individually created their own designs after observing several examples by the way of the above two presentation modes. These designs were evaluated by two evaluators in terms of similarity, originality, and completeness. Statistical analysis showed that no significant difference was found in similarity and originality between the two groups, whereas the designs of the physical group outperformed those of the pictorial group in terms of completeness. This finding indicated that the two groups showed the same degree of design fixation, as multiperspective pictures presented most of the form information of the physical object. The results suggest that when instructing design novices, it is essential to control how to present design examples at different stages of the design process.

## Introduction

A design example(s) can provide designers with knowledge and ideas that constitute the source of a new design [1,2]. However, exposure to design examples may lead to design fixation, resulting in a decrease in the quality of the created design. The negative influences conjured by design examples may be more prominent with design novices [3–5]. The degree of design fixation and the quality of new designs vary with different modes of presenting the design example. A pictorial example is the most common type of medium to convey visual information encountered during the design process, and it is presented more frequently than a physical example [6] because the picture is easier to access and can display form information. Of the studies focused on the influence of various presentation modes of examples on idea generation and quality, the topic of pictorial examples constitutes a large portion of the design research [7–10]. With respect to physical examples, studies have mainly focused on interactivity, because physical examples exceed pictorial examples in the design outcomes in the field of engineering design [6,11–15]. These studies adopted one picture to display information in pictorial format, which could allow designers to understand how the example works in engineering design studies. However, in the study of product design appearance, it is difficult to show the overall form of an example with a picture capturing only one side of an object. This one-picture approach leads to an increase in the gap of the information presented by pictorial and physical examples. When form information provided by the physical and pictorial examples are remarkably similar, will the degree of design fixation and quality be different? In this study, we investigated the influence of physical and multiperspective pictorial examples on design quality in appearance design.

### Form information of the examples

Multiperspective pictorial examples present an amount of form information that is similar to that provided by physical examples. However, the latter contains more sensory information. Specifically, physical examples provide more information about three-dimensional space and physical transitions and include tactile information. The surface texture of an object can be perceived through touch [16], which also strengthens awareness of the spatial location and orientation of the object [17,18]. Designers may benefit from experiencing a physical object using various sensory modalities [19]. This difference in appearance information may be reflected in the designer’s evaluation of the appearance of the design examples. Appearance evaluation is a product appearance analysis based on the designer’s experience, including the analysis of the relative geometry between product features and each product feature [20,21]. Therefore, we propose the following hypothesis:

Hypothesis 1: Participants exposed to physical and multiperspective examples will differ in their appearance evaluation of the design examples.

### Design fixation

Exposure to design examples helps a designer in producing innovative ideas but also leads to design fixation, which may limit design ideas and hinder innovative design activities. Design fixation is defined as blind adherence to a set of ideas or concepts that limits the output of conceptual design [22]. Mentally, design fixation is associated with obstructed memory retrieval [23]. However, the specific mechanisms underlying design fixation are not well understood, and it is difficult to measure design fixation directly. In the present study, design fixation is often reflected in the design results, which may appear identical or similar to the design examples [24–28]. It is common to assess design fixation through the degree to which the design examples are copied [29,30].

Researchers have found that the degree of design fixation varies according to different presentation modes of design examples. In a study on pictorial examples, exposure to a set of pictures hinting at the same central theme could lead designers to bring that hint into their own design [10]. The hint is the additional information perceived by designers. In contrast, among computer-aided design (CAD), photograph and sketch representations, CAD representations encourage designers to identify and to copy key effective features of the example into the design results, while photograph and sketch representations show less visual information [31]. On the other hand, providing partial photographs can reduce design fixation compared to full photographs [8], as partial photographs lack some form information that allows designers to search for ways to fill in missing spaces [32]. Thus, we believe that the degree of design fixation may be related to the amount of information presented by the design examples. In this study, the pictures we provided include multiple perspectives of the presented examples, but they provided less information than physical examples, as mentioned above.

To explore the impact of the two presentation modes on design fixation, we propose the following hypothesis.

Hypothesis 2: Designs generated by participants exposed to physical examples will be more similar to the examples compared to those generated by participants exposed to multiperspective pictorial examples.

### Design quality

In addition to design fixation, examples can affect the quantity, novelty, variety and other qualities of design [29,30]. A few studies have addressed the effect of differences in pictorial examples and physical examples on idea generation and quality. The design produced upon exposure to physical examples through visual inspection and product dissection showed less functional novelty and form-based novelty than those produced by 2D [11]. Christensen and Schunn [33] found that physical examples could inhibit analogies to distant domains, leading to a lack of innovation in design. Presenting diverse rich pictures and sketches in the designer’s work environment could result in differences in originality [9]. These studies show that providing more visual information of an example may lead to a design of lower originality.

When dealing with pictures conveying incomplete information, designers attend most strongly to gaps or segments with missing information [8]. These ‘gaps’ can be useful, as they may provide the designer with a certain amount of space in which to explore other design possibilities. Similarly, the information conveyed by pictorial examples is less than that conveyed by physical examples, especially with respect to space transformation. These gaps may encourage designers to continue searching for missing information until they feel that the gap has been filled [34], resulting in more original designs. In addition, pictorial examples facilitate the participant’s activation of abstract information, which is not associated with examples in long-term memory [35,36], which may be the basis of an original idea.

To explore the impact of the two presentation modes on the originality of designs, we propose the following hypothesis.

Hypothesis 3: Designs inspired by multiperspective pictorial examples will be more original than those motivated by physical examples.
Hypothesis 4: Participants exposed to multiperspective pictorial examples will perceive their inspiration more positively than those exposed to physical examples.

Physical examples are interactive, allowing designers to identify problems during interaction or assist the designer in attending closely to the details of the object. Prior studies have found that physical examples could help designers reduce cognitive load through externalizing ideas [37], aid thinking about complex interactive processes [38], promote understanding of the examples [39,40], and gain better comprehension of the solution space [41,42]. Furthermore, physical examples tended to stimulate participants produce more elaborate idea representations [43]. Therefore, we propose the following:

Hypothesis 5: Designs generated by participants interacting with physical examples will be more complete than those created by participants viewing multiperspective pictorial examples.
Hypothesis 6: Participants exposed to physical examples will pay more attention to product details than those exposed to multiperspective pictorial examples.

## Experiment

We conducted an experiment to investigate the influence of physical examples and multiperspective pictorial examples on form design. In this experiment, participants were asked to design an object under specific design requirements. We manipulated the presentation mode of the examples.

### Design requirement

Product appearance design is the most commonly encountered type of design task for industrial designers. In this design experiment, the participants were asked to design the product appearance according to two criteria, e.g., originality (different from the examples and existing products in the market) and completeness (designed as completely and with as much detail as possible). In addition, the participants could generate more than one design within the given time. We did not require the participants to consider material, color, or internal structure in their designs, which are more difficult to access from the pictorial examples.

### Design examples

We adopted a handheld garment steamer, with which the participants were unlikely to be familiar, as the design object of this experiment to reduce the influence of objects encountered in daily life [44] and to prevent the participants from retrieving images in their minds on the basis of having seen or used the same kind of object. The handheld garment steamer is quite different from a full-size garment steamer in appearance. The former is often used for business trips, while the latter is more commonly used at home and with which participants might be familiar.

We collected 22 handheld garment steamers from online stores at Amazon.com and Taobao.com. These items were classified into four groups based on their similarity of appearance. We purchased the representatives of each group. By reading the product manuals and interacting with the object, we learned that the handheld garment steamer is composed of seven parts: steam outlet, water inlet, switch, handle, power supply, water tank, and identification information. The identification information includes graphic illustrations related to usage, such as the water tank calibration and the rotary direction of the water inlet, not a logo.

**Fig 1:**
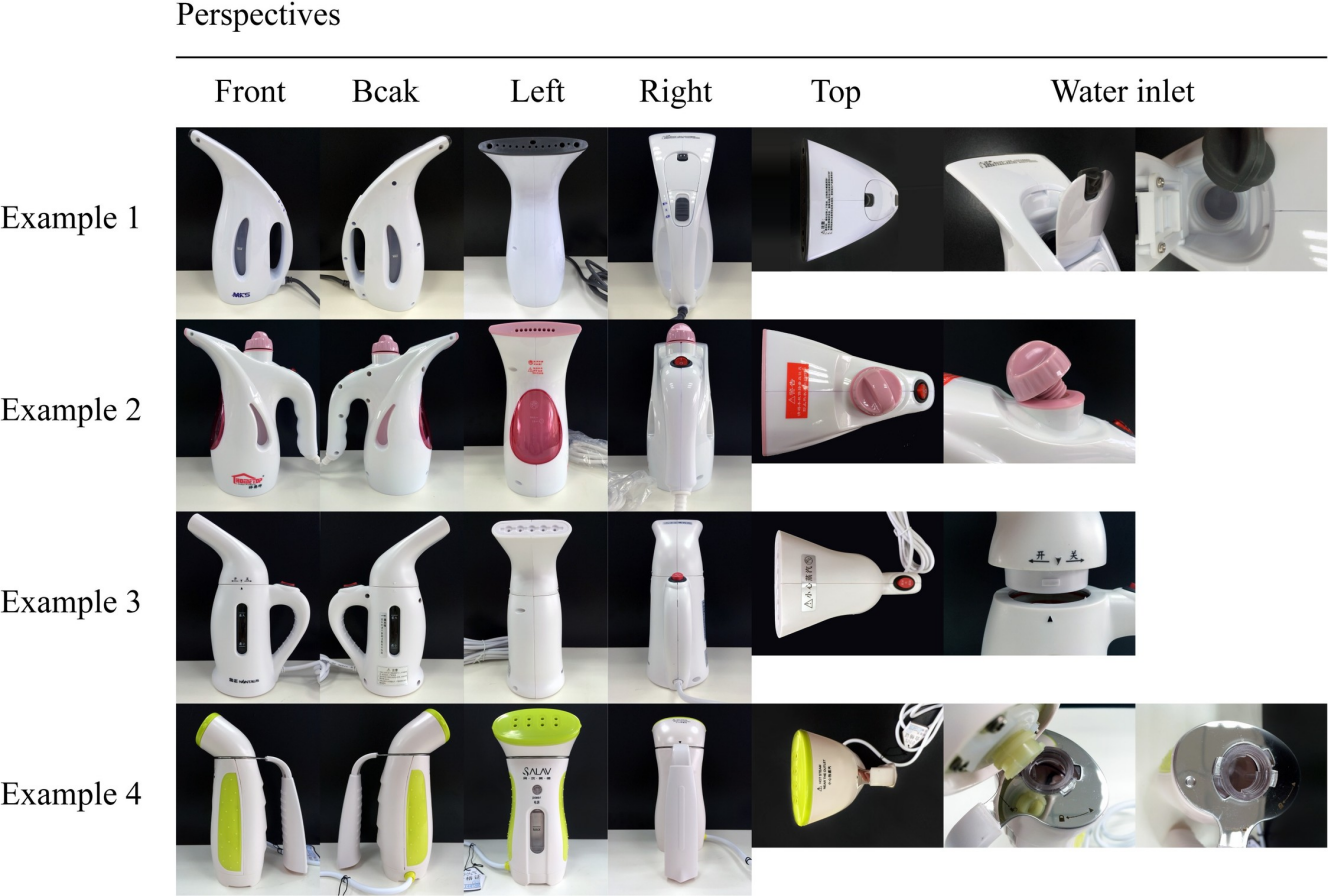
Multiperspective pictorial examples.

These handheld garment steamers were used as physical examples for the physical group. Then, we photographed these examples with the same background, using the same perspective and attempted to show the seven components as clearly as possible. The pictures shown in Fig 1 were used as multiperspective pictorial examples for the pictorial group. The pictures of each example contained the following perspectives: front side, back side, left side, right side, top side, and detail of the water inlet, as some water inlets were visually complex.

### Participants

To ensure that all participants were unfamiliar with the examples, we asked them whether they had seen or used a handheld garment steamer before the formal experiment. As a result, only participants who had never used the design object were included in the study. Ultimately, a total of 60 participants (35 women and 25 men, M = 20.72, SD = 0.96) took part in this experiment. These participants were all in their junior year at university majoring in industrial design. The participants were randomly divided into two groups, i.e., a physical group and a multiperspective pictorial group. Each participant received 30 RMB as a reward for their involvement. All the participants provided written consent and were treated in accordance with national and international norms governing the use of human research participants. The Art and Design Institute of Zhejiang Sci-Tech University granted permission for the performance of the experiment.

### Experimental process

The experiment was conducted in a closed room. We provided the participants with paper, a pencil, an eraser, and design examples according to their group. The experiment was divided into three stages: the description of the experiment, presentation of the examples, and sketch design.

The participants were first required to read the experimental description and complete Questionnaire I. The experimental description introduced the design object, design requirement, general structure and usage of the product, and experimental process. Questionnaire I collected basic personal information, e.g., sex and age.

Then, the participants were shown either physical or multiperspective pictorial examples, according to their group assignment, and were asked to complete Questionnaire II, and the items are shown in Table 1. The participants evaluated the items through a seven-point Likert scale, where 1 meant strongly disagree and 7 meant strongly agree. The questionnaire was designed to familiarize the participants with the form of the examples and then to test hypothesis 1. The time required to complete Questionnaire II was 15 minutes.

**Table 1:** Questionnaire.

Questionnaire II		
Integral Shape	IS-I	I think the integral shape of Example 1/2/3/4 is very innovative.
	IS-B	I think the integral shape of Example 1/2/3/4 is very beautiful.
Local Shapes	LS-I	I think the steam outlet/water inlet/switch/handle/power supply/water tank/identification of Example 1/2/3/4 is very innovative.
	LS-B	I think the steam outlet/water inlet/switch/handle/power supply/water tank/identification of Example 1/2/3/4 is very beautiful.
Questionnaire III		
Inspiration	IP	The physical/multiperspective pictorial examples inspired me during my design process.
Attention	AT	The physical/multiperspective pictorial examples made me pay more attention to the design of product details during my design process.

After completing Questionnaire II, the participants started sketching a design. The participants were able to interact freely with the physical or multiperspective pictorial examples during the design process. The time allocated for completing the sketch design was 40 minutes. At the end of the sketch design, the participants were required to mark the design sketches they planned to submit, and thus abandoned some sketches that they thought imperfect. Eventually, the participants were asked to complete Questionnaire III, which is presented in Table 1, and their responses were used to assess whether the two exposure methods had different impacts on the participants’ subjectivity for testing hypothesis 4 and 6. The questionnaire also adopted a seven-point Likert scale, where 1 meant strongly disagree and 7 meant strongly agree.

### Design evaluation

We obtained a total of 81 designs. The participants in the physical group produced 41 designs while the participants in multiperspective pictorial group produced 40 designs. To evaluate the designs, we invited two senior doctoral students majoring in industrial design to complete a third-party evaluation.

This study used similarity, originality, and completeness indexes to test hypothesis. These measurements are commonly used in design studies [8,11,28,45]. The similarity index was commonly adopted to measure design fixation by comparing the proposed designs with the given examples. The originality index was used to measure the originality of the proposed designs through comparing the proposed designs with the products existing in the market. Obviously, the two indices are different. This is because the designs similar to the given examples are certainly not original, while dissimilar designs (or parts of designs) may exist in the market and thus are non-original. The remaining index was used to evaluate completeness of the proposed designs. In summary, we use the above three indices to verify Hypotheses 2, 3 and 5, respectively.

Considering that the design examples might affect the entire design or only influence some parts of the design, the third-party evaluation was divided into two parts, i.e., the integral shape evaluation and local shape evaluation, including the steam outlet, water inlet, switch, handle, power supply, water tank, and identification information, which are mentioned above.

The evaluation adopted a seven-point scale. Specifically,

1. For the similarity evaluation, a score of 1 indicated that the integral or local shape was completely dissimilar to the four design examples, 2 indicated that the similarity degree was low, 3 indicated slightly lower similarity, 4 indicated neutral, 5 indicated that the similarity degree was slightly higher, 6 indicated high similarity, and 7 indicated very high similarity. No scores were given to missing components.
2. For the originality evaluation, 1 indicated that the degree of originality was very low, 2 indicated low originality, 3 indicated slightly lower originality, 4 indicated neutral, 5 indicated slightly higher originality, 6 indicated high originality and 7 indicated very high originality. No scores were given to missing components.
3. For the completeness evaluation, 1 indicated that the integral shape was very incomplete or the local part had not been drawn, 2 signified that the degree of completeness of the integral shape or local part was low, 3 indicated slightly less completeness, 4 indicated neutral, 5 indicated slightly greater cmpleteness, 6 indicated a high degree of completeness, and 7 a very high degree of completeness.

**Fig 2:**
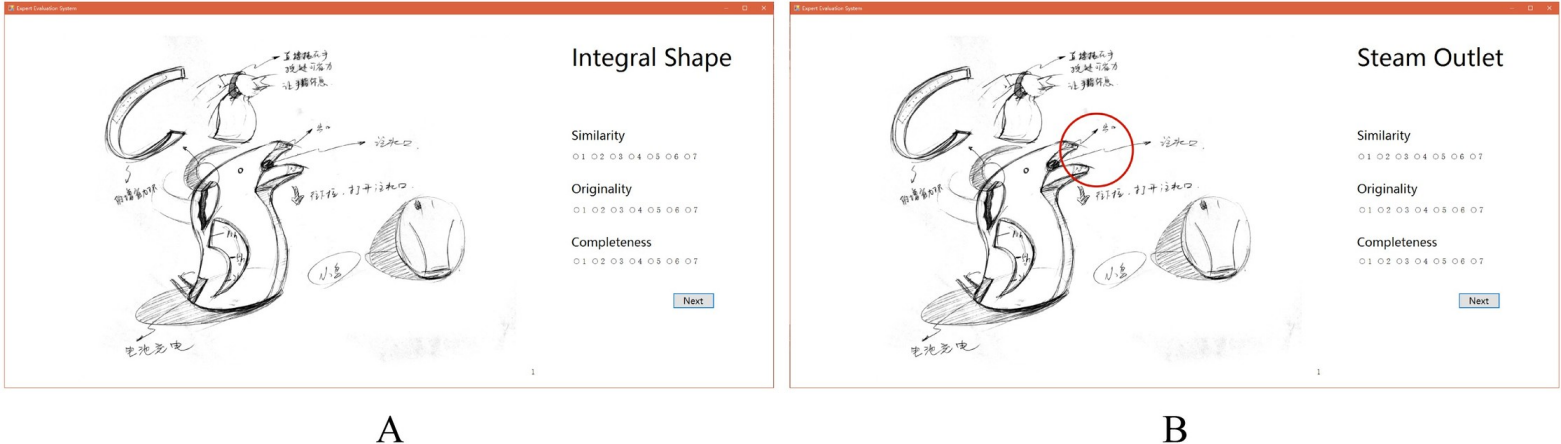
Interface of the Evaluation System (A: Evaluation of integral shape; B: Evaluation of local shapes (steam outlet)).

For the ease of handling of this high number of designs, we developed an evaluation system, as shown in Fig 2, where Fig 2A presents the evaluation of integral shape while Fig 2B shows how we indicated local shapes, which are identified with a red circle for clarification. The designs were randomly presented to both evaluators.

Before the formal evaluation, the two evaluators could observe the four examples in detail to become familiar with them. Then, the evaluators scored the 81 designs in terms of similarity, originality and completeness.

## Results

### Example evaluation by participants

The results of the t-test for Questionnaire II showed that there were significant differences between physical and pictorial groups on the evaluation of Example 4. Specifically, in the evaluation of integral shape, the score for the IS-I of the physical group (M = 5.13, SD = 1.01) was significantly higher than that of the pictorial group (M = 4.17, SD = 1.23), t(58) = 3.23, p<0.01. In the evaluation of local shapes, the score for the LS-I (handle) of the physical group (M = 5.17, SD = 1.12) was significantly higher than that of the pictorial group (M = 4.03, SD = 1.45), t(54.45) = 3.39, p<0.001. This result might be because only the handle in Example 4 can be removed for carrying convenience, while the participants of the pictorial group could not detect this feature. Therefore, hypothesis 1 was partly supported.

### Self-assessment

The results of the t-test obtained for the Questionnaire III responses showed that the AT of the physical group (M = 5.73, SD = 0.94) was significantly higher than that of the pictorial group (M = 5.00, SD = 1.23), t(58) = 2.59, p<0.05. This meant that the participants exposed to physical examples held the view that they paid more attention to the design of the product details. Therefore, hypothesis 6 was supported, while hypothesis 4 was not supported.

### Design quantity

As we did not limit the quantity of designs, some participants generated two or three designs within the stipulated time. Table 2 shows the number of designs generated by the two groups. A chi-square test revealed that there was no significant correlation between the presentation mode of the examples and the number of designs submitted for evaluation (χ2 (2, N = 60) = 0.400, p>0.05).

**Table 2:** Number of the designs generated by the two groups.

	Numbers of designs			Total of designs
Group	1	2	3	
Physical group	21	7	2	41
Pictorial group	21	8	1	40

### Similarity

A consistency check was performed with respect to the scores on similarity given by the two evaluators, where the Cohen’s kappa correlation coefficient was 0.394 (95% CI 0.347–0.441), p<0.001, indicating that their scores had general consistency. Notably, each design was scored individually, while some participants submitted several designs. It indicates that the effects on the participants who generated two or three designs were greater. To reduce the potential biases from the factors associated with these participants, we first calculated the mean scores of each design and further calculated the average of the mean scores of the designs submitted by the same participant. Finally, we obtained average scores of 60 participants for the similarity index.

As the scores might not fit the normal distribution, we used a nonparametric test. The results of Mann-Whitney U tests showed that only one part (the water inlet) showed a significant difference between the physical group (mean of ranks = 35.23) and multiperspective pictorial group (mean of ranks = 25.77) in terms of similarity (U = 308.00, p<0.05). Therefore, hypothesis 2 was partly supported.

**Fig 3:**
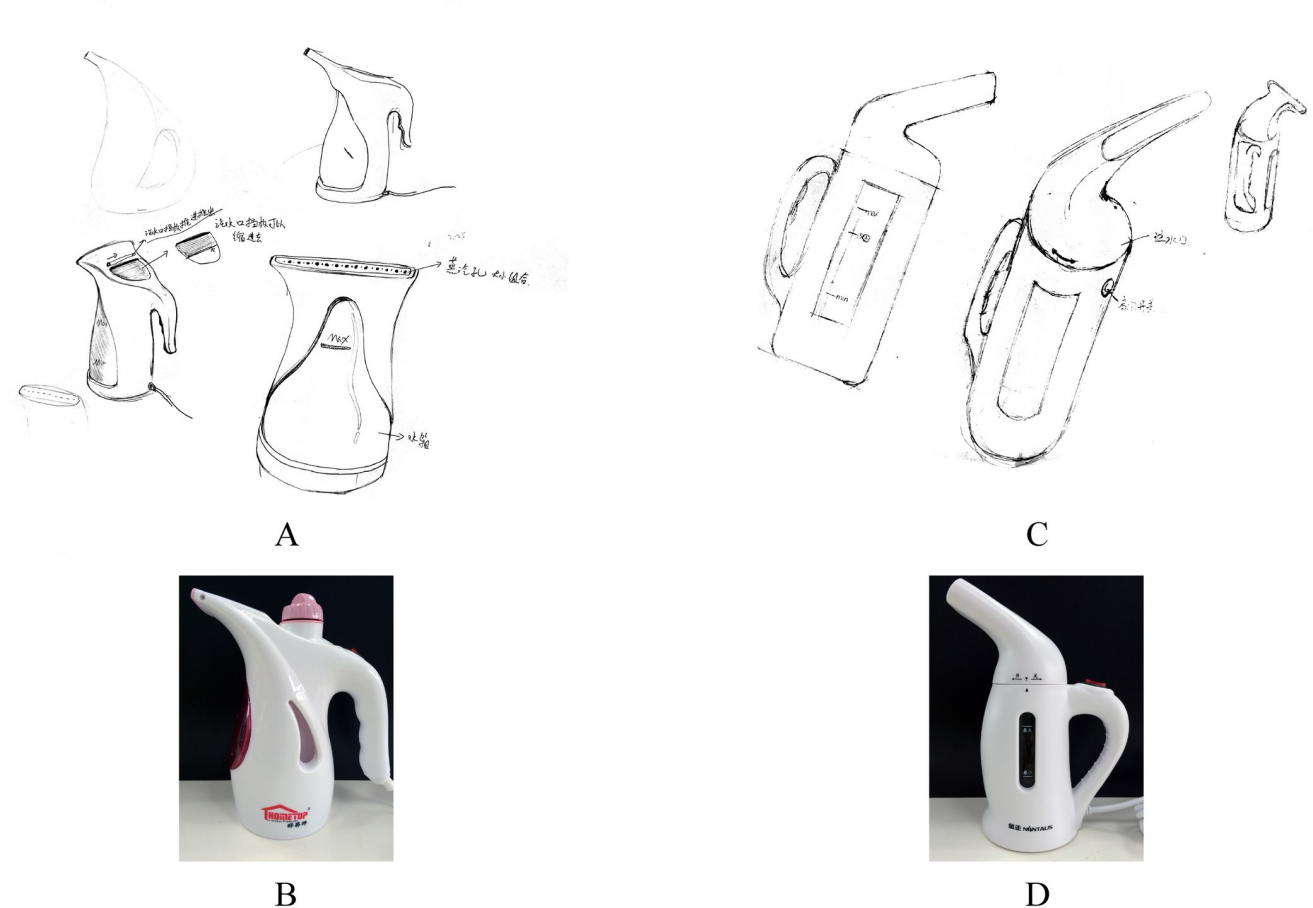
Comparison of the Examples and Designs with High Similarity in the Integral Shape Evaluation (A: A design submitted from physical group; B: Design example 1; C: A design submitted from pictorial group; D: Design example 3).

Fig 3 shows two designs highly similar to the given design examples in integral shape.

### Originality

The Cohen’s kappa correlation coefficient on the originality scale between the two evaluators was 0.442 (95% CI 0.392–0.492), p<0.001, indicating that their scores had moderate consistency. Similarly, we adopted the same procedure described in section 5.4 to obtain unbiased average scores. The Mann-Whitney U test showed that, on average, there was no significant difference between the two groups in terms of originality (p>0.05); therefore hypothesis 3 was rejected.

**Fig 4:**
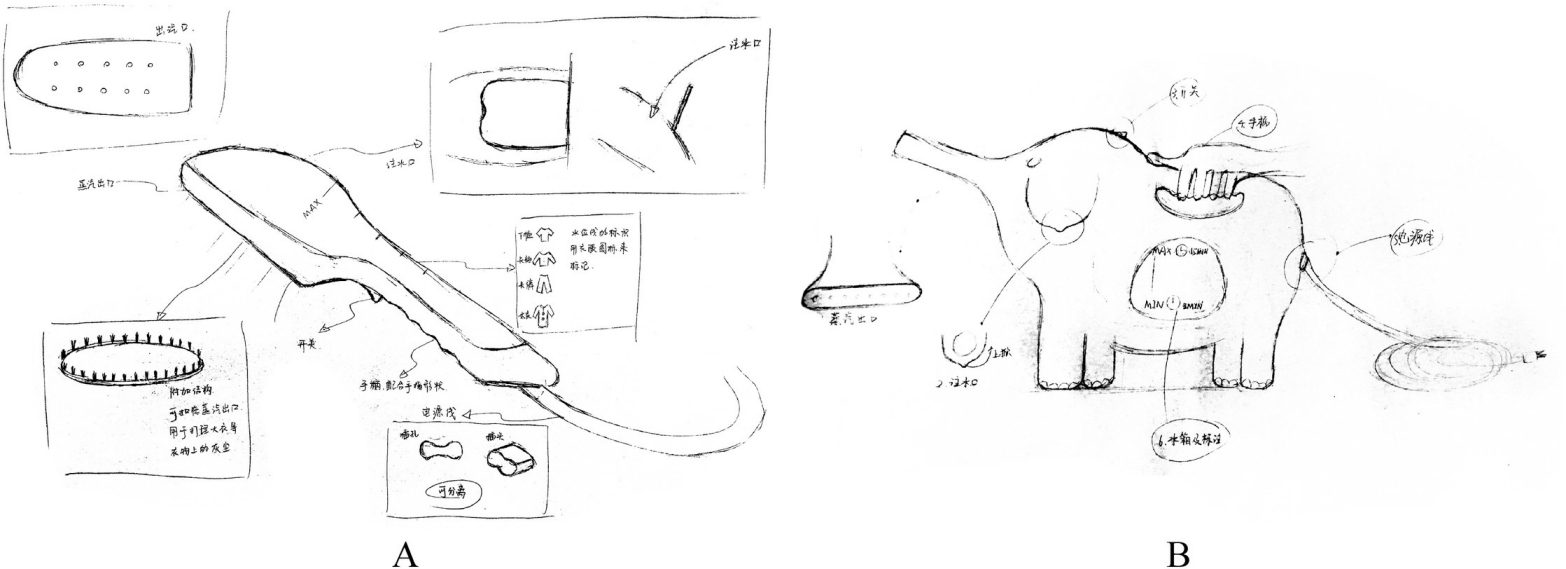
A High Degree of Originality in the Designs of the Two Groups (A: A design submitted from physical group; B: A design submitted from pictorial group).

Fig 4 shows two designs with highly original integral shapes. Through evaluation, two kinds of innovative approaches were revealed. Specifically, in the design representing the physical group shown in Fig 4A, the integral shape was normal, but it was different from the form of the four design examples. On the other hand, in the designs representing the pictorial group (Fig 4B), an existing real object was chosen as a prototype, e.g., an elephant, mouse, dog, bird, or fire extinguisher, and different parts of the selected prototype were mapped to parts of the handheld garment steamer. For instance, Fig 4B shows an elephant chosen as a prototype with its nose, ear and tail designed as the steam outlet, water inlet, and power line, respectively.

### Completeness

The Cohen’s kappa correlation coefficient on the completeness scale between the two evaluators was 0.424 (95% CI 0.374–0.474), p<0.001, indicating that their scores had moderate consistency. We adopted the same method to obtain the average scores of the designs submitted by the 60 participants. The Mann-Whitney U test showed that the integral shape (mean rank of physical group = 35.28, mean rank of pictorial group = 25.72, U = 306.5, p<0.05), steam outlet (mean rank of the physical group = 36.18, mean rank of the pictorial group = 24.82, U = 279.5, p<0.05), handle (mean rank of the physical group = 35.45, mean rank of the pictorial group = 25.55, U = 301.5, p<0.05), and water tank (mean rank of the physical group = 35.3, mean rank of the pictorial group = 25.7, U = 306, p<0.05) indicated significant differences in terms of completeness, which supported hypothesis 5.

**Fig 5:**
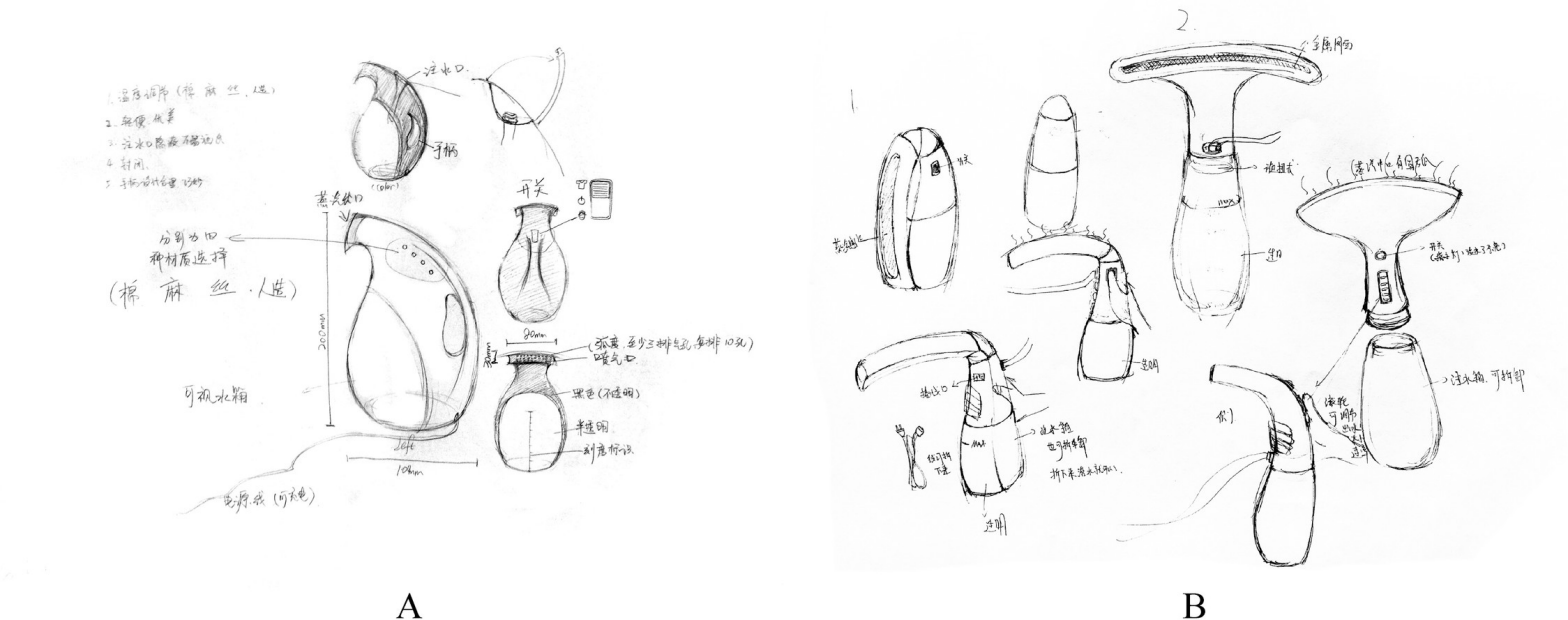
Designs with High Completeness in the Two Groups (A: A design submitted from physical group; B: A design submitted from pictorial group).

Fig 5 shows two designs with high completeness of the integral shape. The participants of these designs used multiperspective and local detail to present their ideas. Further, the design representing the physical group in Fig 5A showed labelled size dimensions.

**Fig 6:**
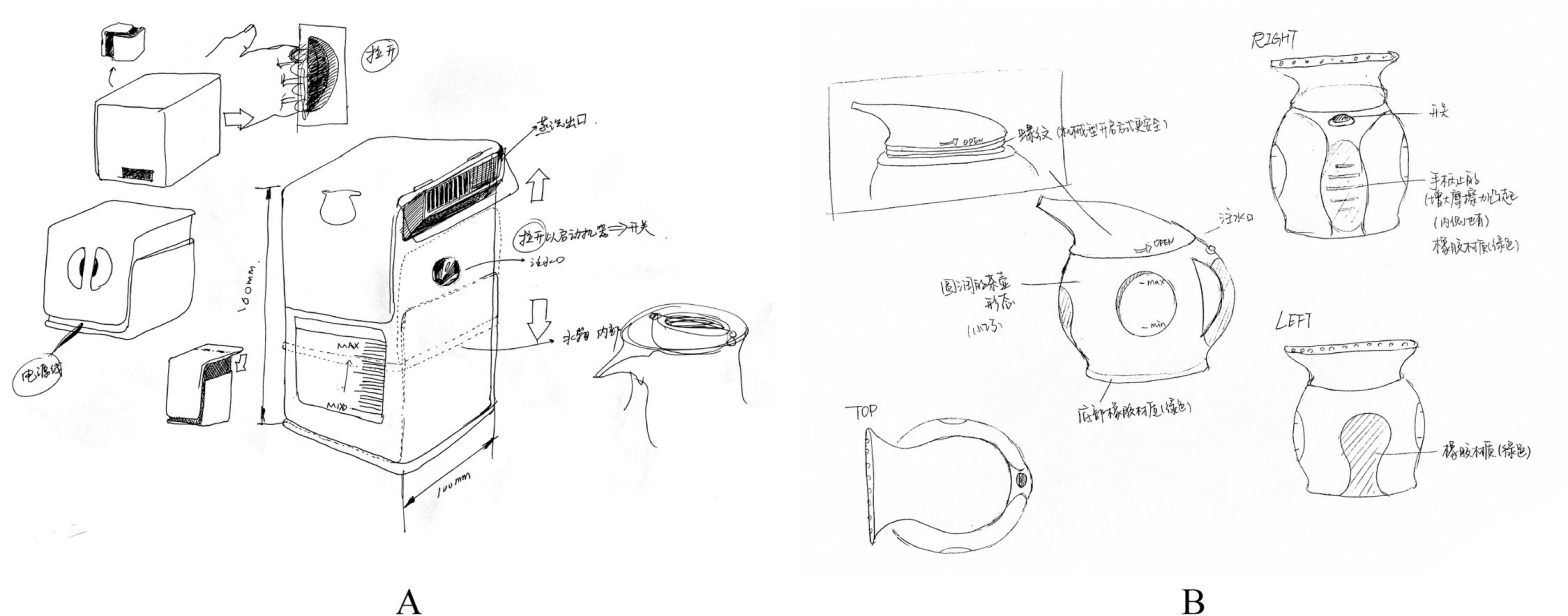
Designs with different Drawing Perspectives in the Two Groups (A: A design submitted from physical group; B: A design submitted from pictorial group).

In addition, the chi-square test revealed that there was a significant correlation between the presentation mode of the design examples and the viewpoints of the drawings (χ2 (1, N = 60) = 4.44, p<0.05). Specifically, the physical group usually used perspective drawing, while the pictorial group preferred a 2D view. For instance, a design submitted from the physical group (Fig 6A) was mainly based on perspective drawing, while a drawing submitted from the pictorial group (Fig 6B) included renderings from the front, back, right, and top perspectives, and the details of the water inlet were also rendered from a 2D perspective.

## Discussion

These analyses reveal that the designs generated by participants exposed to physical examples are not significantly more similar to the examples, than those generated by participants exposed to the multiperspective pictorial examples, except for the water inlet. In other words, the design fixation induced in the participants of the two groups showed a negligible difference. This outcome was because the pictures presented showed almost all of the form information extant in the physical objects. This result is consistent with that of Goel’s [31] study, in which the same information was by provided through a CAD model, photo and sketch, and the designs showed no significant differences in the repeating features of the design examples. Perttula and Sipilä [46] also found that the number of examples presented did not show a respective effect on idea generation. In addition, Viswanathan and Linsey [14] support that designers may fixate on both physical and pictorial examples to the same extent in the field of engineering design. On the other hand, with the exception of the ‘water inlet’, the physical group generated sketches more similar to the design examples than the pictorial group, because the water inlet was the only component that could be observed in detail by disassembling it and was available only to the physical group. Therefore, the physical examples and multiperspective examples performed almost equally in terms of participant’ design fixation.

The designs submitted by both groups also showed no significant difference in terms of originality. Although this result is inconsistent with that of Toh, & Miller [11], it is reasonable since the previous study focused on function design, while in our study, the pictures present the full view of the design examples to reduce the gap in the form information between pictures and physical objects. Notably, originality is not the opposite of similarity. Designs similar to the given examples are certainly not original, while dissimilar designs (or parts of designs) may exist in the market. Therefore, the physical examples and multiperspective examples also performed almost equally in terms of participant’ creativity.

Additionally, the completeness of the designs produced by the physical group was significantly higher than that produced by the pictorial group in integral shape and three local parts, which were more important for understanding the form of the product than other small or standard components. In addition, the drawings submitted by the physical and pictorial groups showed preferences for 3D and 2D perspective, respectively, in the designs. The potential reason is that the pictures that we provided for the pictorial group were mainly in 2D, taken from different perspectives. Hence, the participants in the pictorial group had to make an effort to piece those pictures together to build an integrated prototype in their mind. However, this process may have been more difficult for design novices than it would have been for professional designers. On the other hand, the physical examples allowed the participants to focus on the details of the design because they could perceive the 3D spatial form, which helped the participants with design completeness and was a relatively user-friendlier presentation mode for design novices. Therefore, the physical examples performed better than the multiperspective pictorial examples on design quality.

In summary, physical examples can be more beneficial for design novices than pictorial examples when these presentation modes are used to show examples that provide close-form information. Design educators can manipulate the presentation modes of design examples at different stages of the design process for design novices. Physical examples are suitable for physical contact by design novices, which typically strengthens their spatial perception of the objects, which should be presented after the idea generation stage to refine design sketches. Specifically, design novices can visit factories and exhibitions, through which they can gain some insight from the details they observe. Although physical examples are relatively inconvenient and costly to provide, they are effective stimuli that help designers to learn basic design skills. In the design industry, design factories and enterprises usually encourage designers to use physical models to obtain better understanding [47,48]. Overall, providing design examples is a common method to gain inspiration, and how to make rational use of them is a worthy issue in design education [49].

## Conclusions and limitations

In this paper, we investigated the difference in the influence of physical objects and multiperspective pictures on design fixation and quality. We used several handheld garment steamers as examples. Two groups of design novices observed these physical objects or multiperspective pictures of the objects and then proposed their own designs. By evaluating the designs and comparing them with the examples, we found no significant differences in terms of design fixation (indicated by the similarity index, except for a removable part) or originality. However, observing physical objects can help to improve quality (completeness) and to encourage participants pay greater attention to detail.

Nevertheless, this study can be further improved because of the following limitations. All the participants in the experiment were students majoring in industrial design, which restricted the application of our study results. We hope to involve professional designers in future research.

## Supporting information

S1 Data (XLSX)
